# Imagination of Disease as an Indication in Prescribing

**Published:** 1885-07

**Authors:** Wm. Jefferson Guernsey


### IMAGINATION OF DISEASE AS AN INDICATION IN PRESCRIBING.

DR. WM. JEFFERSON GUERNSEY.

This article has naught to do with ridiculous or fanciful illusions, nor with the delirious hallucinations of snakes, hobgoblins, and the like, but with the supposed existence of *possible* morbid conditions, as suggestive of homœopathic treatment of the disease actually present.

Patients really quite ill state, when first applying for treatment, that they believe so and so to be their malady. If they are in error, and the physician think proper, a simple comparison of the existing symptoms with those of the supposed ailment (call it differential diagnosis, if you choose) will usually satisfy them, and the fancy is dispelled. But with some this idea is so fixed and rooted in the imagination that it cannot by argument be removed. Such a delusion frequently becomes an important "guiding symptom" to the choice of the remedy which upon investigation may be found to possess other proven symptoms closely resembling the pains and sensations of the erring patient.

Take, again, the hypochondriac, who is haunted by the imaginary existence of some disease, when, were his fancy removed, he would be found entirely free from pain.

Others are fretted and worried into a state of nervous debility by the fear that they *may* contract or develop some specified disease.

The answer,

> "Therein the patient
> Must minister to himself,"

as given by the Doctor to *Macbeth's* inquiry of

> "Canst thou not minister to a mind diseased?"

is not commendable in a homœopathician. Let us rather reply in *Macbeth's* own words:

> "And with some *sweet*, oblivious antidote,
> Cleanse the stuffed bosom of that perilous stuff
> Which weighs upon the heart."

(May the writer be forgiven for emphasizing the word sweet!)

Our repertories furnish us with names of many remedies suitable to hypochondriasis; in fact, those remedies having the general condition are too many to enumerate here, but some few of these are pointed and may be dwelt upon. For instance, *Plumbum* imagines that he has some incurable disease;
*Raphanus*, that he has an unrecognizable one;
*Can. ind.*, that he is delirious;
*Bryonia*, that he will become so;
*Artemisia abrotanum*, that he is having softening of the brain;
*Atropinum*, that his blood does not circulate well;
——Also, that he has epilepsy;
*Alcohol*, that he has hydrothorax;
*Phosphorus*, the same;
*Veratrum alb.*, that he has cancer;
——That he is blind;
——That he is dumb;
——That she is pregnant;
*Natrum-phos.*, that he would have typhoid fever;
*Ignatia*, that he cannot walk.

As to apprehensions, many remedies fear insanity, and quite a number apoplexy.

*Calc.-carb.*, *Paullinia pinnata*, and *Tarentula* all dread consumption;
*Aralia* dreads some lung disease;
*Bovista* fears the contagion of some disease (not stated);
*Asafœtida*, brain softening;
*Chelid.*, *Calc.-carb.*, and *Staph.*, that his health is ruined;
*Asafœtida*, paralysis;
*Chelid.*, pneumonia;
*Sumbul.*, vertigo;
*Lac-caninum* and *Lachesis*, heart disease; and
*Lachesis*, the cholera.

And may not this last-named remedy act as a prophylactic to the cholera in persons having an undue fear of it?

It may not be foreign to the point in question to refer to the notions of the *friends* of patients as suggestive of treatment. Suppose the case to be that of a child, perhaps eighteen months old. It has just recovered from bronchial catarrh and its mother calls at your office for medicine for the "*worms*." I think I am safe in asserting that this programme is repeated in at least seven or eight cases out of every ten after this trouble in children of that age or younger. Why is this? Because the little one has swallowed the mucus which would have been expectorated by an older child, and this indigestible substance has had precisely the effect upon the stomach that worms would, and is followed by a like result. Explain this to the mother so that she will refrain from using hurtful "worm medicines," but also assure her that if the child has any worms your medicine will remove them; for the voiding of just one little harmless fellow will gratify her, very much to your chagrin. Certain it is, however, that swallowed mucus will cause worm symptoms, and while *Cina* is not by half a hundred the only remedy for helminthiasis, it is, *par excellence*, the medicine for the case just mentioned.

It is well to listen with at least one ear to the diagnosis of the patients' friends, for if you can assure them of their correctness it will gain you their friendship, and if wrong, you can thus avoid too bluntly contradicting what they had previously told the sick one, and by wresting from them the pleasure of saying, "I told you so," you possibly gain ill-will, which may tempt them to do more harm between your visits than you can do good at them.

Again, as with the Cina case, remedies, or at least questions, leading to the discovery of new indications may follow.

If I knew of any drug homœopathic to the *imagination* that one had "*malaria*," I would see to it that every physician in the country was supplied.

But why dwell longer on this matter? It simply teaches the importance of little things which Hahnemann long ago discovered: that that which may appear as the veriest trifle to others will oft suggest the homœopathic and only true remedy for the sick.

